# Digital Pathology-Based Morphometric Analysis of *Saccharomyces cerevisiae* Effects on Splenic Cell Populations in Broiler Chickens

**DOI:** 10.1007/s12602-025-10736-7

**Published:** 2025-09-27

**Authors:** Elena S. Santana-Trujillo, Jennifer D. Salazar-Rincón, Bryan S. Sánchez-Beltrán, Gustavo González-Paya, Angel Cruz-Roa, Julieta E. Ochoa-Amaya

**Affiliations:** 1 Universidad de los Llanos, Villavicencio, Colombia; 2 Facultad de Ciencias Agropecuarias y Recursos Naturales, Universidad de los Llanos, Villavicencio, Colombia; 3 Facultad de Ciencias Básicas e Ingeniería, Universidad de los Llanos, Villavicencio, Colombia; 4 Research, Group On Pathology of Domestic and Wild Animals, Universidad de los Llanos, Villavicencio, Colombia

**Keywords:** CD4^+^, CD3^+^, CD8^+^, CD163^+^, CD20^+^, CD25^+^, Probiotic, Broilers, Immunity, Spleen, Digital pathology

## Abstract

*Saccharomyces cerevisiae* (SC) is a beneficial probiotic for poultry, serving as a natural alternative to antibiotics by promoting biological synergies that enhance animal health and productivity. This study aimed to evaluate the effects of SC on splenic immunomodulation in broiler chickens by characterizing and quantifying immune cell populations—including T lymphocytes (CD3^+^, CD4^+^, CD8^+^, and Treg CD25^+^), macrophages (CD163^+^), and B lymphocytes (CD20^+^)—per mm^2^ of spleen tissue using immunohistochemistry (IHC). Two treatment groups were compared: a control group (CG; *n* = 8) without SC and an experimental group (PG; *n* = 8) supplemented with SC at 10^7^ CFU·g^−1^. Splenic tissue sections were digitized at 20 × magnification using a MoticEasyScan Infinity 60 slide scanner. Immune cell density and positivity percentages (CD3^+^, CD4^+^, CD8^+^, CD20^+^, CD25^+^, and CD163^+^) were quantified using QuPath digital pathology software. SC supplementation significantly reduced cell density and positivity percentage of CD3^+^ and CD4^+^ T lymphocytes vs. CG and positivity percentage of CD25 cells in PG vs. CG. Supplementation with SC in broiler chickens significantly altered splenic immune cell morphometry, particularly in regions containing CD3^+^, CD4^+^, and positivity in CD25^+^, while no effects were observed on cell density or positivity in CD8^+^, CD20^+^, and CD163^+^ macrophages. Additionally, digital pathology proved effective in enabling precise morphometric quantification of immunohistochemical expression in digitized whole slides.

## Introduction

Probiotics are live microorganisms that, when administered in adequate amounts, confer health benefits to the host [4]. These beneficial microbes enhance immune function by stimulating cytokine production, increasing phagocytic activity, and modulating T- and B-lymphocyte populations—key components of the adaptive immune response [109]. In poultry production, probiotics have been widely utilized for over 50 years as growth promoters, feed supplements, and prophylactic agents against disease [5]. Among these, *Saccharomyces cerevisiae* (SC), a probiotic yeast, has demonstrated significant potential in improving avian health and immunity.

Probiotics exert their beneficial effects in poultry through multiple mechanisms, including the maintenance of normal intestinal microbiota via competitive exclusion and antagonism of pathogens. Additionally, they modulate host metabolism by enhancing digestive enzyme activity while reducing detrimental bacterial enzymes and ammonia production, ultimately improving feed efficiency and nutrient digestion. Probiotics also stimulate both local and systemic immune responses. Beyond immunological effects, they contribute to gut health by reinforcing the intestinal mucosal barrier, promoting mucus secretion, and regulating intestinal motility [37, 67, 115].

The avian immune system comprises a sophisticated network of organs, cells, and molecular mediators that collectively provide defense against pathogens [75]. Its architecture includes primary and secondary lymphoid organs, such as the thymus, bursa of Fabricius, and spleen. During the embryonic stage, undifferentiated cells migrate from the yolk sac to the bone marrow, thymus, and bursa of Fabricius [38, 47]. While the spleen serves as a site of granulopoiesis in young chicks, it matures into a crucial antigen-processing center in adult birds [47]. The thymus and bursa of Fabricius play indispensable roles in adaptive immunity by directing the differentiation of progenitor cells into functional T and B lymphocytes [11, 38]. These mature lymphocytes subsequently populate secondary lymphoid tissues, including the spleen, cecal tonsils, Harderian gland, and mucosa-associated lymphoid tissue [47].

The spleen consists of a connective tissue capsule and trabeculae supporting red pulp and gray pulp, the latter containing germinal centers with a central arteriole and dendritic cells. The red pulp primarily contains CD8^+^ T cells, along with fewer CD4^+^ cells, while the gray pulp includes periarteriolar lymphoid sheaths with CD3^+^ T cells (some co-expressing CD4^+^). The ellipsoid zone—equivalent to the marginal zone—harbors B cells [55, 87].

Lymphocyte surface markers are proteins that serve as immunological identifiers, detectable through antibody recognition. The activation and differentiation of T lymphocytes (TL) are governed by signals from the immune microenvironment, including metabolic stimuli [59]. As central mediators of cellular immunity, TL play critical roles in adaptive immune responses [39, 108].

The CD3 + cell is a critical component of signal transduction, where TCR binding to an antigen triggers a response that activates T lymphocytes. Since the CD3^+^ complex is present in all T cells, it plays a vital role in signal transduction prior to antigen responses [100]. Activation of TLR receptors by microbial products induces signaling pathways that stimulate innate immune responses and inflammation [34]. Probiotic yeast can enhance TLR-type receptors and modulate local mucosal immune responses [99].

T lymphocytes (LTs) are functionally divided into distinct subpopulations that orchestrate adaptive immunity. CD4^+^ T helper cells play a central role in immune coordination by activating other immune cells and facilitating antibody production [36, 109] whereas CD8^+^ cytotoxic T cells and B cells promote immunological memory formation [1, 104].

CD4^+^ T cells differentiate into seven major functional subtypes: TH1, TH2, TH9, TH17, TH22, and regulatory T cells (Treg) [59, 89]. Among memory CD4^+^ populations, TH1 cells specifically produce interferon-gamma (IFN-γ) [59]. Treg cells, identified by their CD4^+^ expression and capacity to recognize MHC class II antigens, are characterized by their production of interleukin-10 (IL-10), transforming growth factor-beta (TGF-β), and the transcription factor Foxp3^+^ [59, 89]. The differentiation of naive CD4^+^ T cells into Foxp3^+^-expressing Tregs [89] is driven by short-chain saturated fatty acids, such as butyric acid and lactate, produced by intestinal anaerobic bacteria [7]. These Treg cells play crucial protective roles, and their abundance can serve as an indicator of immune system health [45, 80].

B lymphocytes express both CD20^+^ and surface B cell receptors, which consist of membrane-bound immunoglobulins [70]. Within the spleen, B cells are primarily found in germinal centers where they mediate antibody production [77]. Beyond their humoral immune functions, B cells play crucial immunoregulatory roles by modulating inflammatory responses, controlling hypersensitivity reactions, and maintaining T cell homeostasis [13]. As key components of the adaptive immune system, B cells recognize antigens in lymphoid organs and provide long-term immunological protection through their differentiation into memory B cells capable of responding to recurrent infections [106].

The spleen and lymph nodes receive extensive sympathetic innervation, particularly in T cell zones [45, 93]. Sympathetic modulation of immune responses depends on the expression of functional catecholamine receptors by immune cells. Among these, the β2-adrenergic receptor (β2AR) serves as the predominant adrenergic receptor expressed across multiple immune cell populations, including dendritic cells, macrophages, and both CD4^+^ and CD8^+^ T lymphocytes, as well as B cells [40].

The immune system comprises two complementary arms: innate and adaptive immunity. Innate immunity serves as the body’s first line of defense, providing rapid but nonspecific protection against pathogens. A key cellular component of this system includes CD163^+^ macrophages, which mediate pathogen clearance through phagocytosis and microbial killing. In contrast, adaptive immunity generates highly specific responses after a lag period, orchestrated by T and B lymphocytes. Notably, CD8^+^ T lymphocytes play a critical role in eliminating virus-infected and malignant cells, primarily acting in secondary lymphoid organs such as the spleen [89, 117].

Macrophages play a pivotal role in bidirectional crosstalk between the immune and nervous systems [22] and function as specialized phagocytic cells of the innate immune system [79]. These cells detect pathogens through surface pattern recognition receptors, which bind to microbe-associated molecular patterns (MAMPs). Upon recognition, pathogens are internalized via phagosome formation and subsequently degraded through lysosomal fusion. In avian species, dietary supplementation with *Saccharomyces cerevisiae* enhances macrophage phagocytic activity through β-glucan-mediated immunomodulation [44] while also boosting antimicrobial responses in mononuclear cells and neutrophils [65].

Antigen exposure in avian species triggers coordinated humoral and cellular immune responses. This process begins when antigen-presenting cells (e.g., macrophages) process and present antigens to lymphocytes, initiating a cascade of immunologic events. B lymphocytes subsequently differentiate into plasma cells, while T lymphocytes proliferate into functionally distinct effector subsets. These responses rely on intricate intercellular cooperation mediated by cytokines—molecules that regulate immune cell functions [90].

Probiotic administration represents an effective strategy for modulating avian immune responses to pathogens and vaccines [91]. *Saccharomyces cerevisiae* supplementation demonstrates immunomodulatory potential, enhancing splenic T lymphocyte activity and systemic immunity in poultry [120]. This probiotic strain enhances B cell-mediated immunity by stimulating lymphoid tissue development and promoting B cell diversification and maturation within splenic germinal centers [2]. Beyond its effects on adaptive immunity, *S. cerevisiae* fermentation products improve both humoral and innate immune parameters while maintaining immunologic balance in broilers [23].

Anti-inflammatory cytokines drive the expansion of regulatory T cells (Tregs), which suppress effector T cell activity through immunomodulatory mechanisms (Hua, 2010). These specialized lymphocytes, identified by their constitutive expression of CD25^+^ and FoxP3^+^, prevent excessive immune activation [88] while maintaining critical immunologic functions: self-tolerance preservation and adaptive immune response regulation [40]. The consistent expression of CD25^+^ and FoxP3^+^ not only defines the Treg lineage but also serves as reliable biomarkers for quantifying these cells in avian immunological studies.

Traditional immunohistochemical (IHC) quantification has relied on manual, subjective evaluation of a limited number of microscopic fields (e.g., 10 fields at 10x, 20x, or 40 × magnification). While useful, this approach is constrained by the time-intensive nature of expert visual analysis and the limited tissue area that can be practically assessed at higher magnifications (e.g., 40x). These limitations may introduce sampling bias and reduce the representativeness of results [52].

To enable objective morphometric analysis, this study incorporates digital pathology into the workflow. Whole-slide imaging (WSI) scanners digitize entire tissue sections at high resolution [15], facilitating computational analysis of the complete sample rather than selected fields. Digital pathology—a discipline focused on managing and analyzing digitized tissue samples [68]—enables the application of automated image analysis, computer vision, and artificial intelligence algorithms [29, 84]. This approach provides more objective, reproducible, and precise IHC quantification, including comprehensive evaluation of positively labeled cell proportions across entire regions of interest for multiple antigens.

Given the limited research on the effects of *Saccharomyces cerevisiae* supplementation in broiler chicken diets on immunological status and immune response, this study investigated its influence on CD3^+^, CD4^+^, CD8^+^, CD20^+^, CD25^+^, and CD163^+^ cells, assessing their density and positivity in the splenic parenchyma using immunohistochemistry (IHC) and digital pathology. The evaluation was conducted within a digital pathology framework, enabling whole-slide digitization of IHC samples and automated quantification of both expression (positivity) and cell population density for each target antigen.

### Objectives

The following are the objectives of the study:

- To assess the immunomodulatory effects of *Saccharomyces cerevisiae* supplementation on splenic immune cell populations in broiler chickens through comprehensive immunohistochemical characterization and digital pathology quantification.
- To quantify both cell density (cells per mm^2^) and percentage positivity of key immune markers:
  o T lymphocytes (CD3^+^, CD4^+^, and CD8.^+^)
  o B lymphocytes (CD20.^+^)
  o Regulatory T cells (CD25⁺)
  o Macrophages (CD163.^+^)

Per 1 mm^2^ of spleen section, assessed by immunohistochemistry and digital pathology.

## Materials and Methods

This study was conducted at the Barcelona campus of the Universidad de los Llanos, located 4 km from Villavicencio, Meta, Colombia. The work was performed in two laboratories: the Histopathology Laboratory (School of Animal Sciences, Veterinary Medicine and zootechnics Program, Faculty of Agricultural Sciences and Natural Resources) and the Software and Specialized Computational Infrastructure Laboratory (AdaLab) (Room 312 A, 3rd floor, Einstein Building, School of Basic Sciences and Engineering). The campus lies at 420 m above sea level, with an average temperature of 28 °C, annual rainfall of 4050 mm, and relative humidity of 85%.

### Experimental Work

Biological samples from the spleen were obtained from paraffin blocks containing tissues of broiler chickens used as experimental specimens. These chickens were supplemented with the yeast *Saccharomyces cerevisiae* as part of an earlier institutional project titled: “Uso de harina de cayeno (*Hibiscus rosa-sinensis*) y cajeto (*Trichanthera gigantea*) más probiótico *S. cerevisiae* sobre los parámetros productivos y de digestibilidad en pollos de engorde.” The study included two dietary treatments: T1 (control) (CG; *n* = 8), which lacked *S. cerevisiae*, and T2, supplemented with *S. cerevisiae* in the concentrate at a concentration of 10^7^ CFU·g^−1^ of the probiotic in the experimental feed (PG; *n* = 8).

Tissues obtained from 45-day-old Cobb500 chickens were used. The individuals belonging to the study were raised in the same shed and divided into cages, considering the control and SC-supplemented groups. The individuals had water and food ad libitum; they were fed with a commercial starter diet during the first 15 days according to the manufacturer’s instructions and fattening; both the control group and the SC-supplemented group had similar food consumption. Supplementation with the probiotic began on day 15 with 5 days of habituation, starting records on day 20 of its consumption. The probiotic used corresponded to a commercial product; 5 mg of the dry product was administered for each kilogram of the commercial diet, ensuring the concentration of 10^7^ CFU/g of the probiotic in the experimental diet. The records were Taken from day 20 of the individuals’ lives until the end of the experiment on day 45 of the individuals’ lives [71].

Spleen samples were collected for immunohistochemical characterization of T lymphocyte subpopulations (CD3^+^, CD4^+^, CD8^+^, CD20^+^, CD25^+^, and CD163^+^). Tissue processing was performed as follows: paraffin-embedded blocks were sectioned at room temperature using a Leica rotary microtome. Six serial sections were obtained per sample, floated on a 40 °C water bath, and mounted on poly-L-lysine-coated glass slides (Sigma) with three sections per slide. Then, the slides were dried overnight at 37 °C, individually wrapped in aluminum foil, and stored under desiccation at − 20 °C.

### Immunohistochemistry

Immunohistochemical (IHC) staining was performed to identify and quantify lymphocyte subpopulations, including CD3^+^, CD4^+^, and CD8^+^ T cells, CD20^+^ B cells (LB), CD25^+^ regulatory T cells (Tregs), and CD163^+^ macrophages. Whole-slide histology images (WSIs) of spleen sections were acquired using a Motic EasyScan Infinity 60 pathology scanner and analyzed with QuPath open-source software.

The positive cell detection algorithm was calibrated to the correct pixel-to-micron scale and optimized for cell size, staining intensity, and morphological parameters to ensure accurate quantification of cell density and positivity percentage for each marker. Digital image analysis was performed on 20x-magnified WSIs for both experimental treatments, following established immunohistochemical quantification methods [107].

**Fig. 1:**
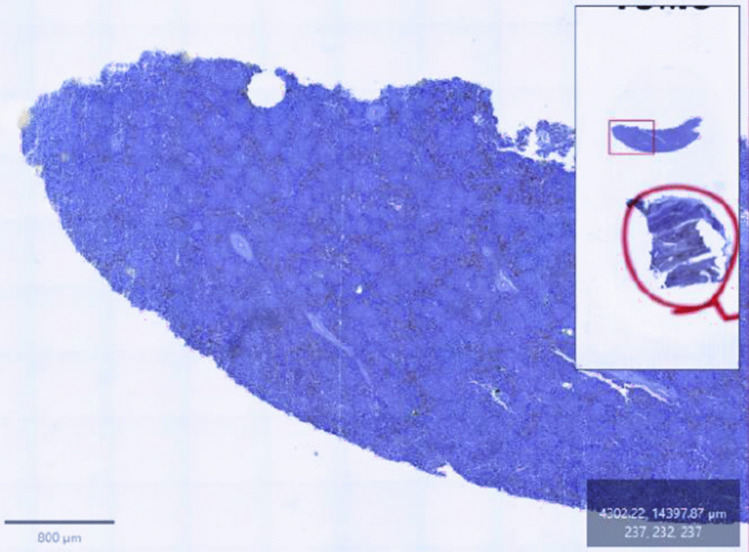
Representative whole-slide digital image of immunohistochemically stained spleen sections visualized in QuPath software. Images were acquired using the Motic EasyScan Infinity 60 slide scanner

Immunohistochemical evaluation was performed using QuPath software to analyze slides from both control and *S. cerevisiae*-supplemented chickens. Scanned images were organized by creating a project folder via the [Create Project] option, followed by image upload through the [Add Images] function. Images were processed using the brightfield H-DAB module for IHC assessment, which detects hematoxylin (H) counterstaining and diaminobenzidine (DAB) chromogen deposition (Fig. 1). This enabled visualization and magnification of the scanned slides as needed.

**Fig. 2:**
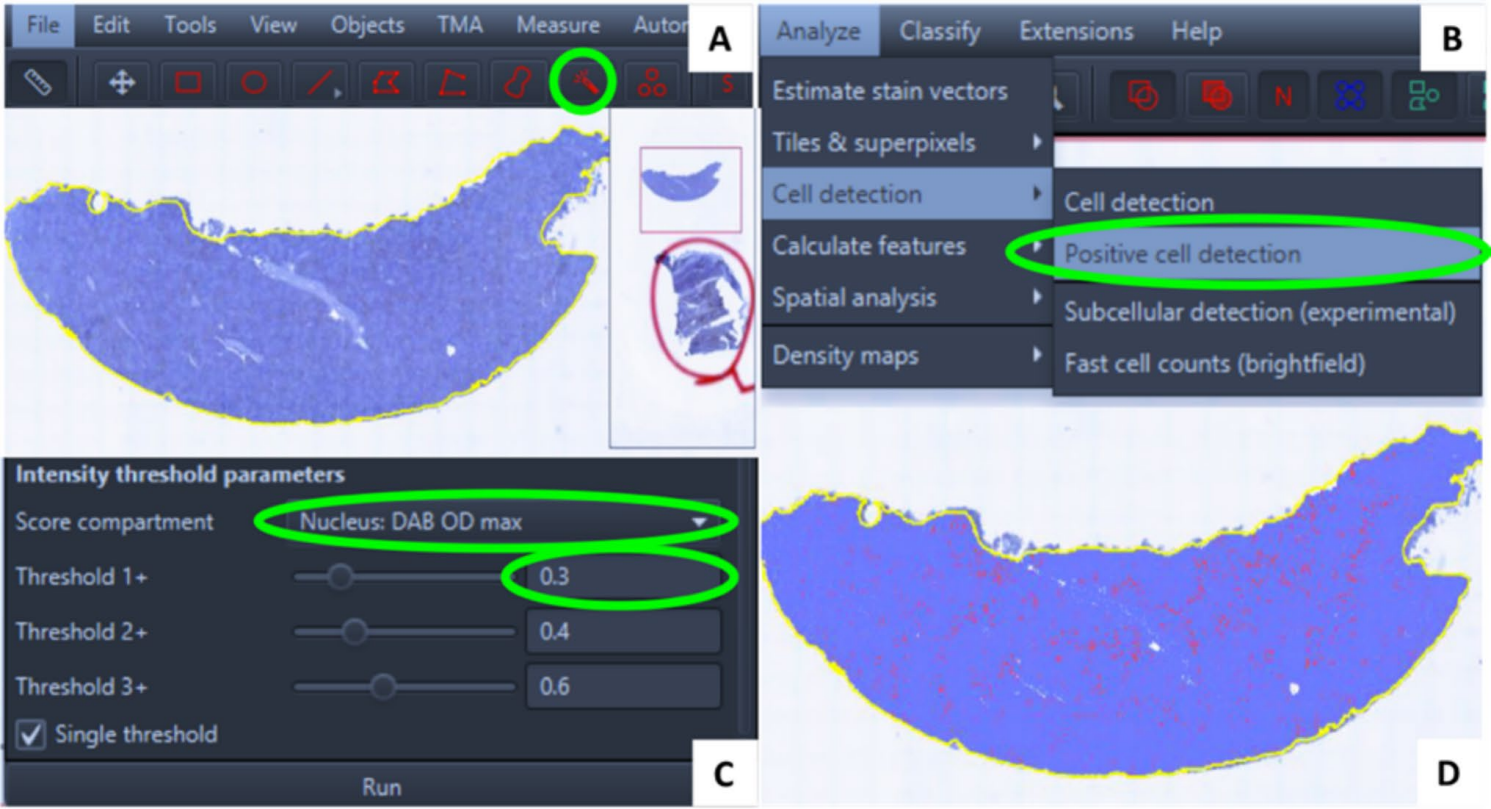
Quantitative immunohistochemical analysis workflow in QuPath software. **A** Tissue region of interest (yellow) delineated using the wand tool (green). **B** Automated positive cell detection module selection (green). **C** Parameter configuration for DAB chromogen intensity threshold (green). **D** Final detection and quantification of target cell populations (red) within the defined tissue region (yellow)

Figure 2 illustrates the step-by-step quantification of immunopositive cells using QuPath software, measuring both morphometric expression (positivity) and cellular density. The analysis began with precise tissue perimeter definition using the wand tool (Fig. 2A). Subsequent steps involved selecting: [Analyze] > [Cell Detection] > [Positive Cell Detection] from the menu bar (Fig. 2B). This opened the parameter settings dialog, where intensity thresholds were configured by: (1) setting the score compartment to [Nucleus: DAB OD max] and (2) adjusting the [Threshold 1 +] value to 0.3 (Fig. 2C). Final positive cell detection was initiated by executing the [Run] command (Fig. 2D).

The QuPath analysis panel displayed quantitative parameters in an interactive interface, with the [Annotations] tab positioned on the left workspace. This tab contained a detailed parameter table organized into [Key] and [Value] columns. The immunohistochemical analysis generated seven key metrics: (1) total cell detections, (2) negative cell count, (3) positive cell count, (4) positivity percentage, (5) positive cell density (cells per mm^2^), (6) measured area (µm^2^), and (7) perimeter length (µm). All quantitative data were systematically compiled and organized using Microsoft Excel for subsequent statistical evaluation.

### Statistical Analysis

Quantitative data were organized in Microsoft Excel and analyzed using parametric statistics. Normality was assessed using the D’Agostino–Pearson omnibus test. For data meeting normality assumptions, group comparisons were performed with an unpaired *t*-test; non-normal distributions (*p* < 0.05) were analyzed with the Mann–Whitney *U* test. All analyses were conducted in GraphPad Prism (v.9.5.1 for macOS 2), with statistical significance set at *p* < 0.05.

## Results

**Fig. 3:**
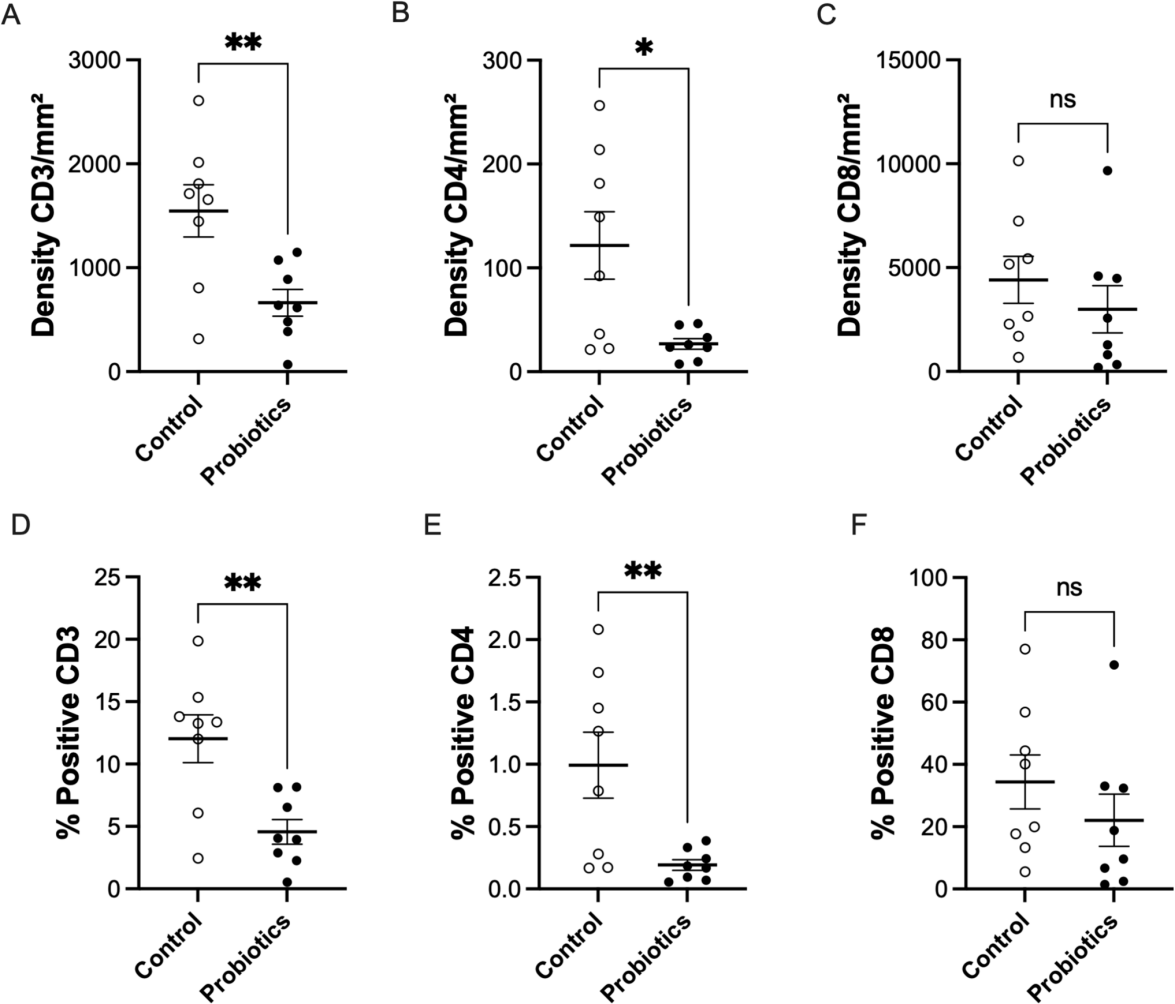
Effects of *Saccharomyces cerevisiae* supplementation on spleen cell density and positivity percentage for C3^+^, CD4^+^, and CD8^+^

Figure 3 presents CD3^+^, CD4^+^, and CD8^+^ cell density and positivity (mean ± SEM) in control (CG) and *S. cerevisiae-supplemented* (PG) groups. The PG group demonstrated significantly lower CD3^+^ cell density (662.9 ± 128.2 vs. CG: 1547 ± 251 cells per mm^2^; *p* < 0.0073) (Fig. 3A) and reduced positivity (4.565 ± 0.9858% vs. CG: 12.02 ± 1.919%; *p* < 0.0038) (Fig. 3D). Similarly, for CD4^+^ cells, PG showed markedly decreased density (26.8 ± 5.108 vs. CG: 121.7 ± 32.45 cells per mm^2^; *p* < 0.0119) (Fig. 3B) and lower positivity (0.1918 ± 0.04299% vs. CG: 0.9928 ± 0.2649%; *p* < 0.0098) (Fig. 3E).

In addition, the unpaired *t*-test analysis of CD8^+^ cell density revealed no statistically significant differences between the probiotic-treated group (*S. cerevisiae*-supplemented, PG) and the control group (CG) (Fig. 3C). Similarly, analysis of CD8^+^ cell positivity percentage showed no significant differences when comparing the control group (CG) with the yeast-treated group (PG) (Fig. 3F).

**Fig. 4:**
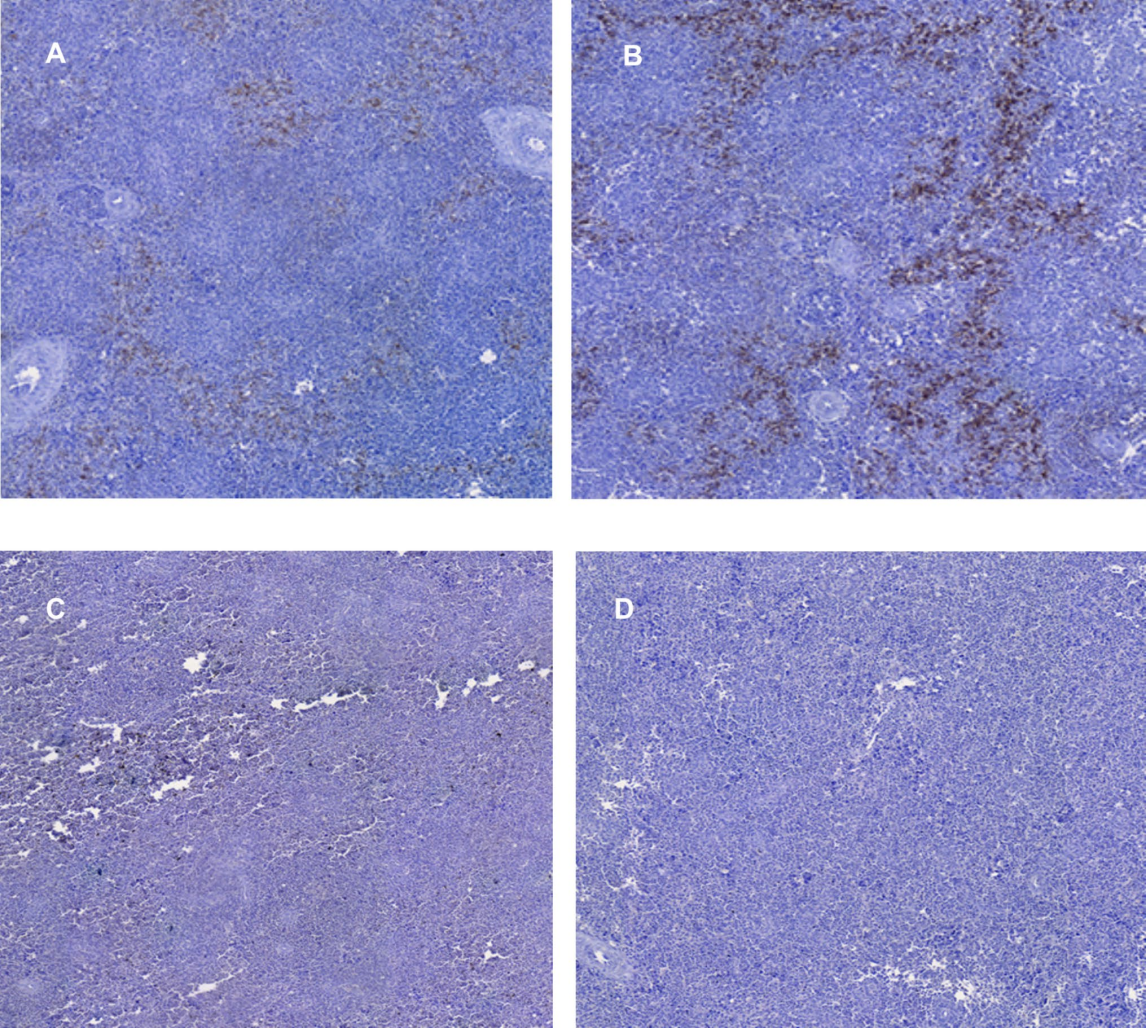

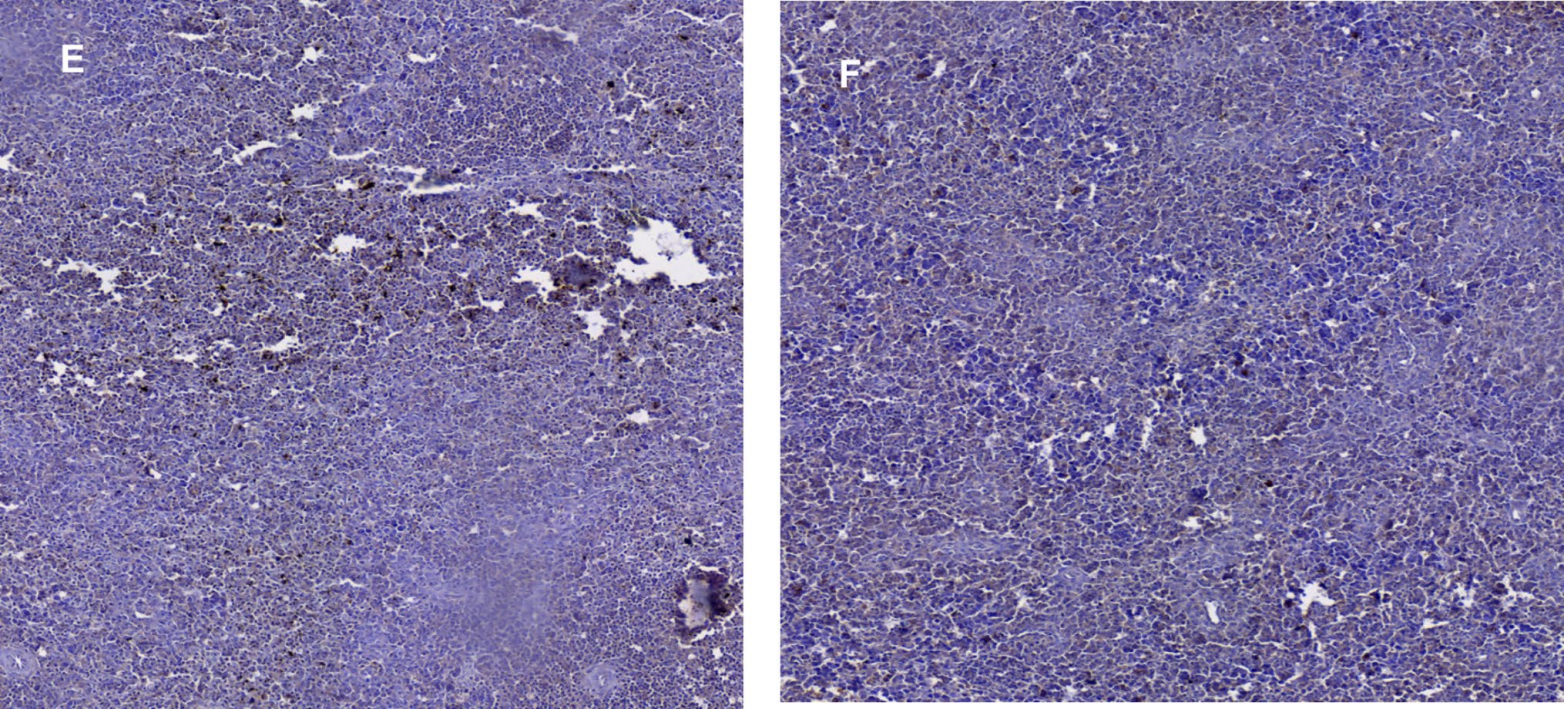
Immunohistochemical detection of CD3^+^, CD4^+^, and CD8^+^ T cells in splenic tissue from control and *Saccharomyces cerevisiae*-supplemented broiler chickens

Figure 4 presents immunohistochemical micrographs of CD3^+^, CD4^+^, and CD8^+^ staining in splenic tissue from both control and probiotic-treated chicken groups.

Figure 4 shows immunohistochemical staining (20x) of splenic sections, with immunostaining observed for CD3^+^ T cells in the control group (A) and probiotic supplemented group (B), CD4^+^ T cells in the control group (C) and probiotic supplemented group (D), and CD8^+^ T cells in the control group (E) and *Saccharomyces cerevisiae* group (F).

**Fig. 5:**
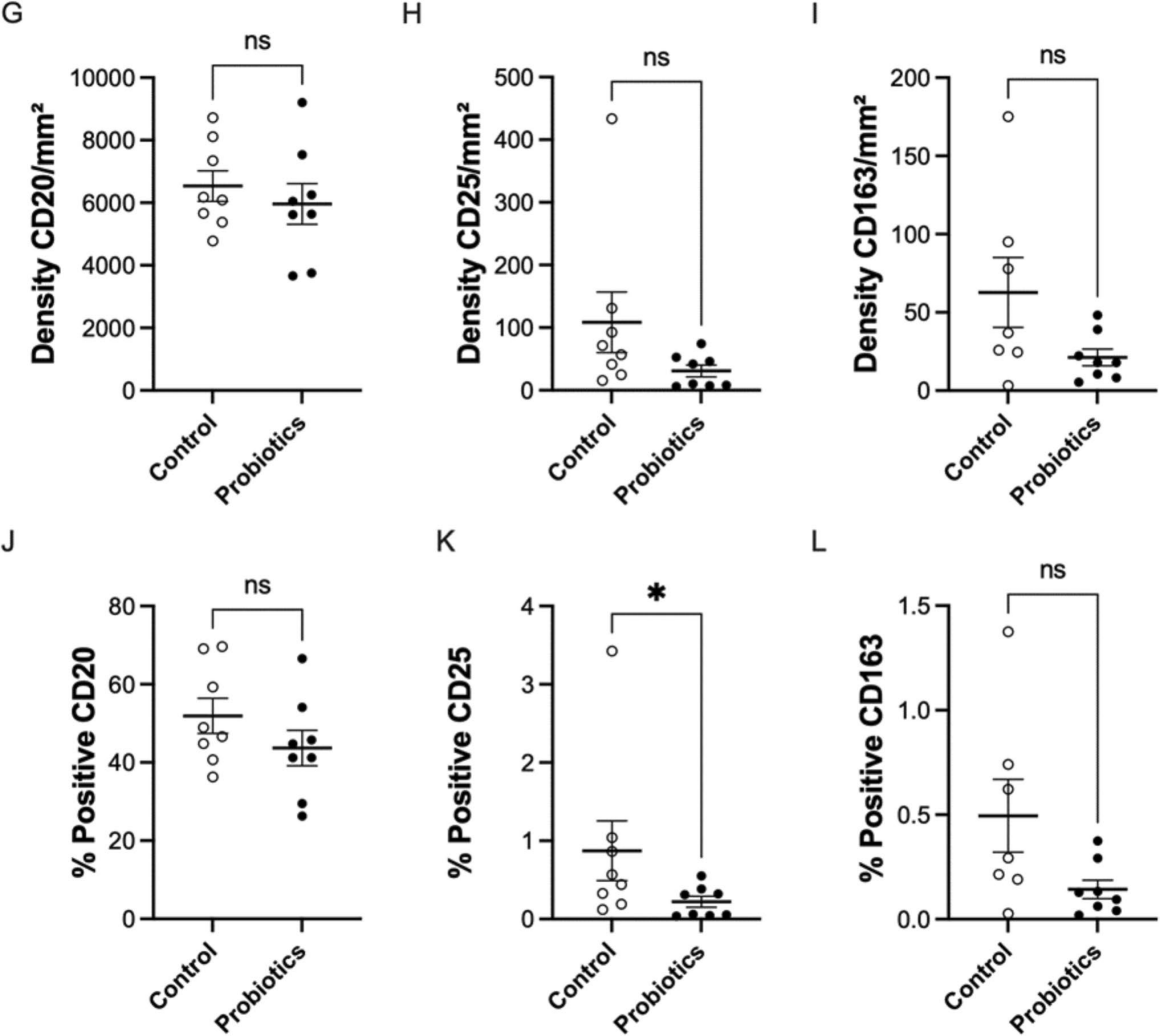
Impact of *Saccharomyces cerevisiae* supplementation on splenic CD20^+^, CD25^+^, and CD163^+^ cell density and positivity percentages in broiler chickens

Figure 5 presents the mean values with standard error for cell density and percentage positivity of LB (CD20^+^), Treg (CD25^+^), and macrophages (CD163^+^) in both the control group and the group supplemented with *Saccharomyces cerevisiae* probiotic yeast. For CD20^+^ cell density per mm^2^, the Mann–Whitney *U* test showed no significant differences between probiotic-treated and control groups (Fig. 5G). Similarly, the unpaired *t*-test revealed no statistical differences in CD20^+^ positivity percentage between probiotic-treated groups and their controls (Fig. 5J).

For CD25^+^ Treg cell density, the Mann–Whitney *U* test showed no significant differences between groups (Fig. 5H). Regarding CD25^+^ cell positivity percentage, the Mann–Whitney *U* test revealed statistical differences (*p* < 0.0281), with the probiotic-supplemented group showing lower positivity (0.2215 ± 0.06981) compared to the control group (0.8730 ± 0.3815) (Fig. 5K).

The Mann–Whitney *U* test showed no significant differences in CD163^+^ macrophage cell density between *Saccharomyces cerevisiae*-treated groups and controls (Fig. 5I). Similarly, the unpaired *t*-test revealed no statistical differences in CD163^+^ cell positivity percentage between control and *S. cerevisiae*-treated groups (Fig. 5L).

**Fig. 6:**
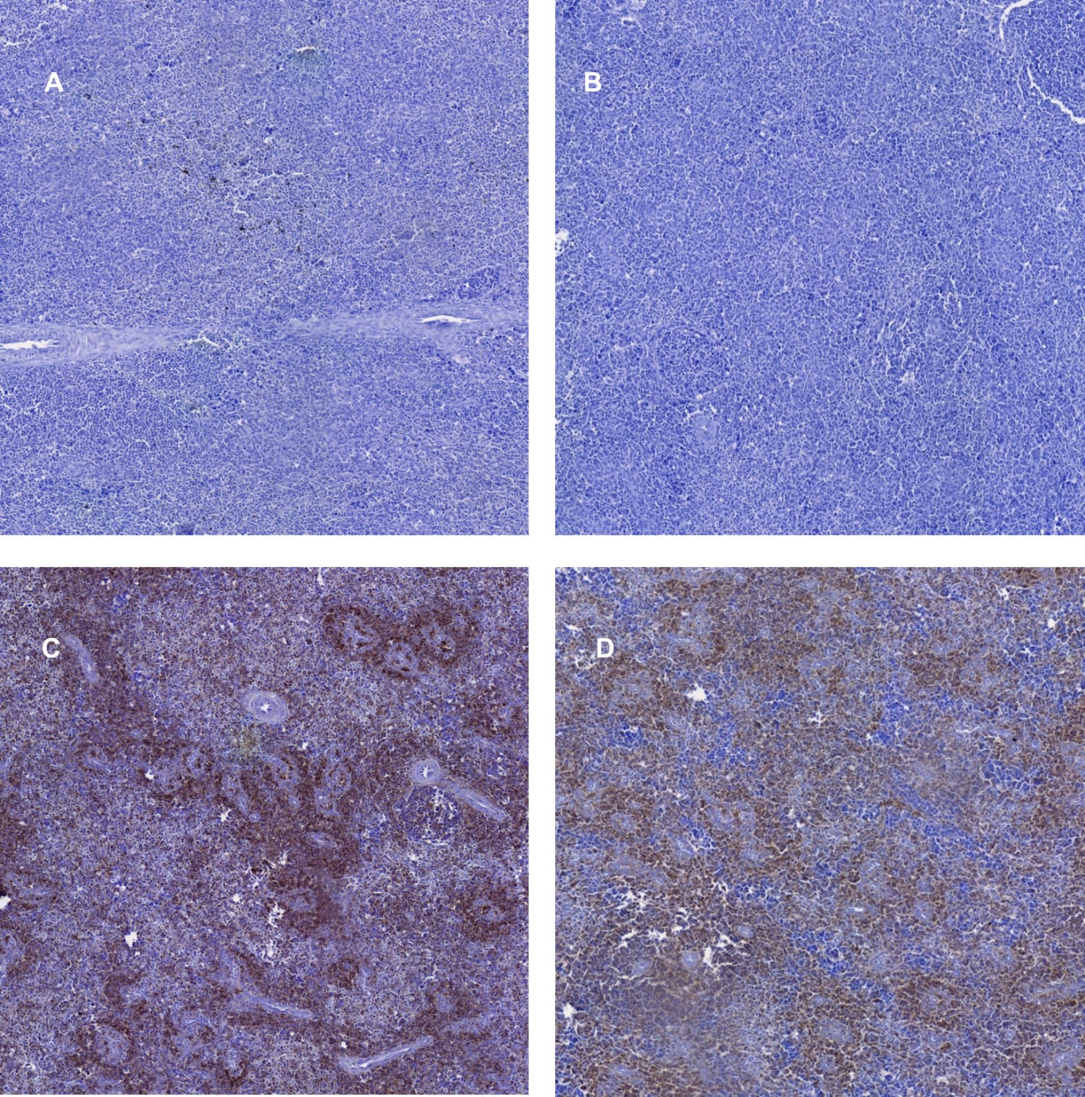

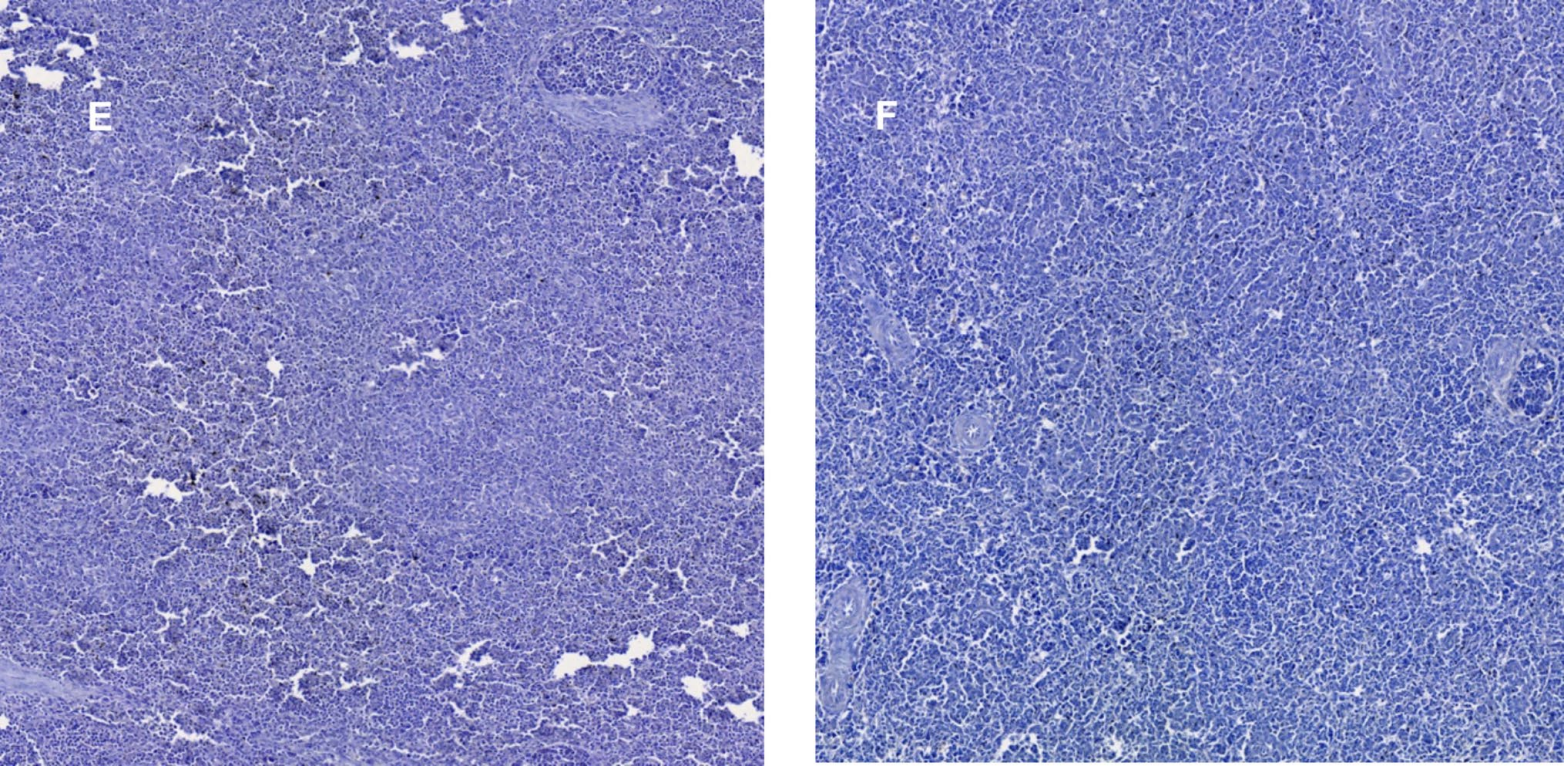
Immunohistochemical staining of splenic immune cell populations in control and *Saccharomyces cerevisiae*-supplemented chickens: CD163^+^ macrophages (**A, B**), CD20^+^ B lymphocytes (**C, D**), and CD25^+^ regulatory T cells (**E, F**)

Figure 6 presents immunohistochemical staining (20x) of splenic sections, showing CD163^+^ macrophages in both the control group (A) and *Saccharomyces cerevisiae*-supplemented group (B). Similarly, CD20^+^ B lymphocyte immunostaining is displayed for the control (C) and probiotic-treated (D) groups, while CD25^+^ Treg lymphocyte staining is shown for the control (E) and supplemented (F) groups.

The *Saccharomyces cerevisiae*-treated groups (PG) showed no significant differences (Mann–Whitney *U* test) in CD20^+^ B lymphocyte density (cells per mm^2^) or positivity percentage compared to controls (CG). Similarly, CD25^+^ Treg cell density revealed no significant differences (Mann–Whitney *U* test) between PG and CG groups.

Table 1 summarizes the above results comparing the control group (CG) and the *Saccharomyces cerevisiae*–treated group (PG) in terms of positive cell density and percentage of positive cells for each IHC marker (CD3^+^, CD4^+^, CD8^+^, CD20^+^, CD25^+^, and CD163^+^), along with their statistical significance. CD3^+^ showed the most consistent differences between CG and PG across both metrics, followed by CD4^+^. In contrast, CD25^+^ was significant only for the percentage of positive cells, but not for density. The remaining markers (CD8^+^, CD20^+^, and CD163^+^) did not show statistically significant differences.

**Table 1:** Quantitative and statistical analysis showing the mean and standard error (SE) for the number of positive cells per area (Density/mm^2^) and the percentage of positive cells (% Positive) for each IHC marker, comparing the control group (CG) and the *S. cerevisiae*–treated group (PG)

Marker	Control (Mean ± SE)	Probiotics (Mean ± SE)	Significance
**Density (mm**^**2**^**)**			
CD3^+^	1546.59 ± 709.83	662.86 ± 362.47	**
CD4^+^	121.65 ± 91.79	26.80 ± 14.45	*
CD8^+^	4412.09 ± 3192.30	2991.33 ± 3,208.33	ns
CD20^+^	6534.31 ± 1385.55	5963.78 ± 1831.11	ns
CD25^+^	108.44 ± 136.56	30.87 ± 26.26	ns
CD163^+^	62.71 ± 59.06	21.19 ± 15.10	ns
**% Positive**			
CD3^+^	12.02 ± 5.43	4.56 ± 2.79	**
CD4^+^	0.99 ± 0.75	0.19 ± 0.12	**
CD8^+^	34.36 ± 24.53	22.05 ± 23.68	ns
CD20^+^	51.93 ± 12.65	43.68 ± 12.84	ns
CD25^+^	0.87 ± 1.08	0.22 ± 0.20	*
CD163^+^	0.49 ± 0.46	0.14 ± 0.13	ns

Statistical significance is indicated as not significant (ns), significant (*), and highlysignificant (**)

## Discussion

Probiotics exert both direct and indirect effects on lymphoid cells, particularly influencing T cell regulation and dendritic cell activity through mechanisms involving antigen presentation and specific immune responses [73]. T cell activation and differentiation are mediated by signals from the immune microenvironment, including metabolic stimuli [59].

In adaptive immunity, controlled proliferation enables clonal expansion of antigen-specific T and B cells, generating both short-lived effector cells and long-lived memory populations. The functionally distinct CD4^+^ (helper) and CD8^+^ (cytotoxic) T cell subsets maintain immunological memory through precisely balanced survival, apoptosis, and homeostatic proliferation. Notably, yeast polysaccharides interact with major histocompatibility complex class II (MHC II) to stimulate cytokine secretion from CD4^+^ T cells, subsequently activating B cells and macrophages to initiate inflammatory, antitumor, and antimicrobial responses [58, 110].

Consistent with Zhang et al. [116], *Saccharomyces cerevisiae* demonstrates cytoprotective effects on avian lymphocytes. Our findings of reduced CD3^+^ and CD4^+^ cell density and positivity percentage suggest an absence of chronic inflammatory or autoimmune processes in chickens supplemented with this probiotic. This immunological quiescence may result from competitive exclusion of pathogenic bacteria in the intestine by *S. cerevisiae*, thereby reducing microbial colonization and disease risk [7, 40, 48].

The coordinated reduction in both CD3^+^ T cells and their CD4^+^/CD8^+^ subsets—consistent with the role of CD3^+^ as a T cell marker—reflects diminished T cell activation and differentiation, aligning with the immunomodulatory effects of *S. cerevisiae* [59]. The reduced frequency and positivity of these cells suggest limited CD4^+^ T cell activation and differentiation, consistent with an absence of autoimmune or inflammatory processes in supplemented chickens [59].

Furthermore, probiotic-fed birds demonstrate significant reductions in pathogenic bacteria, including *Escherichia coli* [56, 92, 116] and *Clostridium* spp. [6]. In addition, probiotic supplementation has been shown to increase populations of beneficial lactobacillus and bifidobacteria [64, 118], collectively indicating improved intestinal health and reduced disease susceptibility through enhanced microbial balance.

Glucans are structural polysaccharides that constitute the primary component of yeast cell walls, including those of *Saccharomyces cerevisiae* [31]. Functioning as biological response modifiers [79], they are recognized as pathogen-associated molecular patterns (PAMPs) by pattern recognition receptors (PRRs) on innate immune cells [81]. Upon ingestion, glucans are processed by monocytes, macrophages, and dendritic cells within intestinal lymphoid tissues before being transported to immune organs such as the spleen, where they are degraded into smaller soluble particles like glucan-1,3 [31].

In turn, 1,3/1,6-β-glucans enhance macrophage phagocytic activity [40] and stimulate lymphocyte proliferation [83]. This occurs through T cell receptor (TCR) signaling and its interaction with major histocompatibility complex (MHC) molecules during antigen presentation [114]. The CD3^+^ complex subsequently transmits activation signals to the T cell cytoplasm, initiating a cascade of biochemical reactions that promote T lymphocyte proliferation [101]. However, such an effect was not observed in the present study, as no increase in lymphocyte population density was detected.

In contrast, Cox et al. [27] observed reduced IL-8 expression in chickens supplemented with *Saccharomyces cerevisiae*-derived β-glucans compared to non-supplemented birds, suggesting immunomodulatory and anti-inflammatory effects [40, 82]. Although cytokine levels were not assessed in our study, flow cytometric analysis of CD3^+^ and CD4^+^ cells revealed a significant reduction in their splenic populations following *S. cerevisiae* supplementation.

These findings align with prior work by our research group, which demonstrated that yeast exposure decreased both the total number of splenic germinal center (NGC) profiles and their density per mm^2^ (NaGC). Additionally, an increase in intercellular spacing—both in sectional (Δ2) and spatial (Δ3) dimensions—was observed within germinal centers. This effect may be attributed to β-glucan-mediated interactions with pattern recognition receptors (PRRs), such as TLR2 and TLR4, on macrophages (unpublished data) [61].

The observed reduction in both density and positivity rates of CD3^+^ and CD4^+^ cells in the *S. cerevisiae*-supplemented group supports the role of probiotics as immunomodulators in poultry, enhancing intestinal health and disease resistance. However, such strain-specific effects cannot be generalized to other strains within the same species [73]. Additionally, prior research indicates that β2-adrenergic receptor (β2AR) activation during CD4^+^ T cell stimulation suppresses proliferation and downregulates IL-2 receptor α chain (CD25^+^) expression via an AMP-dependent mechanism [40].

On the other hand, complex polysaccharide structures called glucans, found in fungal and yeast cell walls, are classified as biological response modifiers (BRMs) [42]. As microbe- and pathogen-associated molecular patterns (MAMPs/PAMPs), β-glucans exhibit immunomodulatory activity by binding to specific receptors (e.g., dectin-1) on immune cells, triggering processes such as phagocytosis. Interestingly, low- and medium-molecular-weight β-glucans—particularly those derived from acid degradation—can suppress macrophage activation by inhibiting free radical and cytokine production [119]. However, under certain circumstances such as inhibition of actin-mediated phagocytosis of large β-glucan particles [62].This antagonistic effect on β-glucan receptors may explain the observed reduction in macrophage-positive areas in the *Saccharomyces cerevisiae*-supplemented group. Uptake of β-glucan in the small intestine and activation of innate and adaptive immune cells of Peyer’s patches, lymph nodes, and systemic organs. Orally administered β-glucans can be either absorbed through M cells or through binding to the projected tips of dendritic cells (DCs) in the follicle-associated epithelium (FAE) of Peyer's patches, and subsequently bind to dectin-1 and TLR2. The macrophages or DCs engulf β-glucans and fragmented β-glucans (FBGs) are secreted in the lymph nodes. FBGs, like soluble β-glucans, bind to dectin-1, but are unable to activate macrophages and DCs [9]. Interestingly, Dectin-1-activated pDCs promote Th2-type T cell responses, while Dectin-1-activated mDCs suppress Th2 responses [54].

Macrophages serve as the primary immune responders by phagocytosing these structures [79] and processing them into smaller 1,3-glucan-containing particles. These particles are then transported to immune organs such as the spleen, where they prime immune cells for heightened antimicrobial and inflammatory responses [31]. For instance, particulate β-glucan enhances immune function by activating antigen-presenting cells, which improves tumor phagocytosis and promotes TH1 responses along with cytotoxic T lymphocyte activity. Additionally, soluble β-glucan binds to complement receptor 3 (CR3^+^), triggering antibody-dependent cellular cytotoxicity and phagocytosis to aid in tumor elimination [119].

Oral administration studies demonstrate the systemic immunomodulatory effects of β-glucan. In mice, β−1,3-glucan supplementation (80 mg·kg^−1^) significantly boosted splenic B and T cell proliferation in response to mitogens compared to controls [95]. Similarly, oral β-glucan supplementation activated cytotoxic T lymphocytes, B cells, and macrophages in murine models [28], confirming its broad immunostimulatory capacity across multiple lymphocyte populations.

Contrary to our findings, Quintín et al. [78] propose that glucan primes innate immune cells through progenitor chromatin marker reprogramming (trained immunity). This is supported by enhanced antimicrobial and inflammatory responses in monocytes/macrophages following glucan exposure, mediated through TLR and dectin-1 activation. Yitbarek et al. [113] observed upregulated splenic TLR2/TLR4 expression in chickens fed 0.02% yeast-derived supplements by 42 days. Similarly, Cox et al. [26] reported increased iNOS activity, enhancing macrophage pathogen destruction through superoxide generation. However, Callol et al. [19] documented dose-dependent phagocytosis increases (42.7–94.1%) in *Totoaba macdonaldi* cells with glucan stimulation. In avian studies, Cheng et al. [25] found *Schizophyllum commune*-derived β−1,3-glucan enhanced macrophage chemotaxis but did not affect mitogen-induced lymphocyte blastogenesis.

CD8^+^ cytotoxic T lymphocytes play a crucial role in both antigen-specific and non-specific cell-mediated immune responses. As key responders to systemic infections, splenic CD8^+^ T cells rapidly activate upon encountering antigen-presenting cells (APCs) like dendritic cells, proliferating and differentiating into effector cells to eliminate infected targets. The spleen’s secondary lymphoid architecture facilitates these interactions, enabling CD8^+^ T cells to coordinate with CD4^+^ T cells and B cells (CD20^+^) for effective immune responses [31].

The maintained CD8^+^ cell density and positivity of CD8^+^ cells in *Saccharomyces cerevisiae*-supplemented groups suggests absence of endogenous antigens, compared to controls. This study demonstrates that splenic macrophages (CD163^+^) and CD8^+^ T cell populations and positivity of both cells remained unchanged after treatment with *S. cerevisiae.*

The PRRs reported for β-glucan include dectin-1, TLR, scavenger receptors (SRs), and langerin [119]. While both macrophages and dendritic cells can phagocytose yeast-derived particulate β-glucan, they likely utilize distinct pathways—macrophage phagocytosis being entirely dectin-1 dependent [119]. Notably, these phagocytic and pro-inflammatory receptors share common intracellular signaling molecules and ligands (including β-glucan), suggesting potential functional interactions between different receptor types [103, 119].

Consistent with our findings, Shanmugasundaram et al. [89] observed that yeast cell-wall probiotic supplementation (0.1–0.2%) in unchallenged broiler chickens did not significantly alter CD4^+^ (helper) or CD8^+^ (cytotoxic) T lymphocyte counts in cecal tonsils and spleen compared to non-supplemented controls.

The lack of significant differences in macrophage and CD8^+^ T cell density suggests an absence of stress or disease proliferation. Previous findings by our research group [8, 76] demonstrated that probiotic supplementation enhances intestinal defenses through multiple mechanisms including increased goblet cell density, elevated mucin production, intestinal epithelium thickening, and mucosal layer expansion. These collective modifications strengthen innate immunity by simultaneously inhibiting pathogen colonization and promoting beneficial microbiota associated with gut health [50].

However, contrasting with our results, Qureshi [79] reported increased CD4^+^ and CD8^+^ cells in porcine intestinal intraepithelial lymphocytes following 0.02% β-glucan supplementation for 8 weeks. Similarly, Gao et al. [41] observed elevated CD4^+^ and CD8^+^ T lymphocytes in peripheral blood, spleen, and intraepithelial lymphocytes, along with increased cecal tonsil IgA, in broilers supplemented with 0.25–0.5% autolyzed *Saccharomyces cerevisiae* during *Eimeria tenella* challenge. These studies collectively suggest that yeast supplementation may enhance host immune responsiveness, even in unchallenged animals.

Our present findings showed that *Saccharomyces cerevisiae* did not generate a decrease in the activity of B cells (CD20^+^) and Treg lymphocytes (CD25^+^) present in the spleen. This conclusion is supported by the absence of decreased density in B lymphocytes (CD20^+^) and Treg lymphocytes (CD25^+^) in supplemented broiler chickens compared to controls. Regarding cell density, no significant change was found in the number of lymphocytes compared to the control groups. Some authors describe that B cells, having an adaptive function, become activated through antigen presentation-mediated stimuli, which would typically lead to higher population density (Yitbarek, 2013). The observed latency in cell numbers suggests the absence of inflammatory processes in the animals.

The Treg cells (CD25^+^) only showed decreased positivity in the germinal centers of the experimental group. We suggest that since the immune system did not appear reactive, there was no need for the regulatory activity typically provided by Tregs. This observation does not necessarily imply any reduction in either the innate or adaptive function of the immune system.

Regarding the other evaluated marker CD25^+^ for Treg cells, studies in probiotic-supplemented birds show Treg differentiation is cytokine-dependent, requiring factors like TGF-β during active immune responses against extracellular pathogens [16]. This explains the stable Treg population observed in absence of inflammation. Although probiotics stimulate immune mechanisms like antigen presentation, they typically do not significantly alter lymphocyte counts compared to non-supplemented groups [24].

The gastrointestinal tract serves as the primary site for probiotic interactions and immune modulation. Probiotics induce structural modifications in intestinal tissue, consistent with Yeşilyurt et al. [112], who reported enhanced intestinal characteristics that improve defense against pathogens. Previous work from our research group demonstrated that *Saccharomyces cerevisiae* supplementation altered duodenal histomorphology, increasing mucus production, which prevents microbial penetration and increasing crypt size [7, 66].

Probiotic interactions with the digestive system directly stimulate intestinal tissue, though their immunomodulatory effects extend beyond local gut responses. As demonstrated by Madej (2020), broiler chickens supplemented with prebiotics and synbiotics showed increased populations of B and T lymphocytes—key mediators of adaptive immunity—in both splenic tissue and cecal tonsils, key cellular components of the adaptive immune system.

The term “immunobiotics” was coined in 2003 to describe probiotics that enhance mucosal immunity by stimulating secretory IgA production, thereby modulating intestinal immune responses and systemic immunity. Some *Saccharomyces* species are particularly valuable as immunobiotics due to their antibiotic resistance — a key advantage over bacterial probiotics [73].

Probiotic supplementation in poultry diets initiates signaling mechanisms through epithelial-microbiota interactions, requiring balanced communication for proper mucosal function. As Britti et al. [14] indicated, gut-associated lymphoid tissue (GALT) development and probiotic-enriched microbiota yield mucosal immunomodulatory effects. Specifically, *S. cerevisiae* exerts beneficial immunomodulatory actions in the digestive tract by competing with pathogens [51].

Popov et al. [74] demonstrated that dietary supplementation with probiotic strains and ferments in 160 broiler chickens activated IL-6 and IL-10 gene expression in the spleen. These cytokines promote immune homeostasis through anti-inflammatory functions, simultaneously enhancing both productive performance and immunological status. IL-10, a pleiotropic cytokine, plays a crucial role in modulating inflammatory responses and maintaining immunological balance by preventing excessive immune activation [21].

While the present study did not evaluate cytokines directly, we assessed Treg (CD25^+^) and CD4^+^ cell populations, which are known producers of IL-10. The observed reduction in both cell density and positivity percentage for CD4 + cells in the *Saccharomyces cerevisiae* (SC)-supplemented group suggests the absence of ongoing inflammatory or active immune processes in these animals.

The immune system responds to foreign agents when pathogens breach physical barriers and evade innate defenses. During this process, phagocytosis and pathogen degradation release molecular signals, including cytokines like IL-1, interferons, and TNFα. These substances drive inflammatory responses, which subsequently activate B lymphocytes (LB) — key cellular components of the adaptive immune system [86].

In healthy animals, immune responses are stimulus-specific and depend on the nature of the affecting agent. Probiotic supplementation alters digestive system structure and cellular composition, providing protective effects while maintaining proportional immune responsiveness to pathogenic challenges [33]. Probiotics promote immunological tolerance in healthy individuals, eliciting minimal inflammatory responses during pathogenic encounters. These effects stem from microbial recognition mechanisms involving both acquired immunological surveillance (developed through lifetime microbial exposure) and innate immune components (Diaz, 2017).

Lymphoid activity reflects immune system function through both immune cell formation and specialized immunological organs. The spleen serves as a primary lymphoid organ, responsible for cell maturation and production of defense cells when immune responses are required [41]. Probiotic supplementation directly influences immune organ development, as demonstrated in broiler chickens where yeast-added feed significantly increased the weight of key immune organs including the spleen, bursa of Fabricius, and thymus [35].

Recent research confirms probiotic-induced splenic enlargement, aligning with findings by Sayed and Mahsa [85]. Their study compared broilers fed conventional commercial feed versus those receiving additional probiotics, revealing a 14% greater spleen weight in the probiotic-supplemented group. These results demonstrate the measurable impact of probiotic supplementation on immune organ development even in commercially fed poultry.

Probiotics interact with various immune cells, including epithelial cells, dendritic cells, monocytes, macrophages, and lymphocytes [10]. The probiotic signaling pathway occurs through pattern recognition receptors (PRRs), including toll-like receptors and protein-like receptors, which recognize pathogen-associated molecular patterns (PAMPs) depending on the interacting microorganism. Following recognition, these receptors modulate signaling pathways to either suppress or enhance specific immune responses [43].

T cell activation and differentiation depend on three key signals, namely, TCR engagement, CD28^+^-mediated co-stimulation, and cytokine signaling. Research by Nakaya et al. [67] revealed that metabolic factors within the immune microenvironment additionally modulate these processes. Probiotic microorganisms modulate immune cells throughout the intestinal mucosa, including B cells and Treg lymphocytes. These cells respond to their local microenvironment, which contains various cytokines and inflammatory mediators that collectively regulate immune system activation. Critically, this environment determines the balance between immune stimulation and Treg-mediated suppression [111].

Probiotic recognition occurs through toll-like receptors, triggering anti-inflammatory cytokine release [35]. This process involves prebiotic oligosaccharides interacting directly with gut-associated lymphoid tissue (GALT) cells and blood monocytes. Through TLR4 binding, these interactions modulate pro-inflammatory responses by inducing IL-10 production, which subsequently promotes Foxp3^+^ cell induction [17]. However, our study found no significant differences in Treg cell populations between *Saccharomyces cerevisiae*-supplemented animals and controls. This contrasts with established literature demonstrating probiotic-induced Treg activation mechanisms, suggesting these pathways were not engaged under our experimental conditions.

Human studies, on the other hand, demonstrate that probiotics produce anti-inflammatory and antioxidant metabolites that downregulate TNFα while upregulating IL-10 activity [30]. Additionally, certain probiotic-enriched microbiota can stimulate interferon-alpha (INFα) production, thereby modulating peripheral lymphocyte and macrophage function [82].

The microbiota communicates with the host through multiple pathways, including endocrine, neuronal, metabolic, and immunological routes. These interactions can influence various organs, with outcomes being either beneficial or detrimental depending on the microbiome’s status [3]. According to Manzano et al. [60], cytokines function as regulators of adaptive immune response activation following stimulation. Tolerance-associated cytokines include IL-12, IL-10, TGFβ, and retinoic acid, while TH1 cytokines mediate active immune responses.

Immunohistochemical markers enable precise cell identification and quantification. For B lymphocytes (LB cells), CD20^+^ serves as a specific marker; these cells function as antigen-presenting cells that can activate CD4^+^ T cells [57]. Treg cells are identified by their CD25^+^ expression, which acts as the high-affinity IL-2 receptor. By sequestering IL-2, CD25^+^ Treg cells limit cytokine availability for effector T cells, thereby exerting immunomodulatory effects [78].

The activation mechanism of distinct cell populations depends on specific stimuli and subsequent signaling cascades that ultimately drive effector cell function and regulatory responses. For regulatory T cells (Tregs), activation initiates when effector T cells secrete IL-2, which directly stimulates Tregs to express Foxp3^+^— the key transcription factor mediating their immunosuppressive function. This regulatory activity ceases upon completion of its physiological role, demonstrating the stimulus-dependent nature of Treg responses. In the absence of activating signals, Tregs enter a state of natural anergy characterized by diminished functional activity [20].

Our findings align with this mechanism, as the absence of antigen presentation in the spleen resulted in no detectable Treg activation signals or micro-environmental support for maintenance/expansion. Consequently, we observed stable Treg numbers with minimal functional activity. This latency state reflects the lack of necessary activation stimuli rather than cellular deficiency. Sustained Treg activity requires TGFβ and IL-2, which promote both expansion and survival. Importantly, since activated lymphocytes produce IL-2, this creates a direct correlation between inflammatory processes and Treg activation [46].

The activation of LB cells involves interaction with TH2 cells, leading to subsequent immunoglobulin release [69]. Research in both human and animal models has shown that adult stages exhibit no significant differences in LB cell populations within peripheral lymphatic organs. However, immunomodulatory effects of LB cells are observed in gut-associated lymphoid tissue (GALT) and peripheral systems when supplementation occurs during early developmental stages, while adult supplementation primarily modulates digestive system responses [18]. Notably, probiotic administration during physiologically optimal periods can enhance B lymphocyte differentiation and development [72].

Finally, there are some limitations to consider of this research using digital pathology for morphometric analysis of IHC. Although the scanner provides high-resolution imaging, the optical depth of field, pixel-micron scale, and calibration constraints may hinder precise measurement of small cellular structures [32], while occasional scanning artifacts or uneven illumination can affect segmentation accuracy [96, 105]. Variability in IHC staining intensity, background nonspecific signals, and suboptimal antibody affinity for avian antigens may further complicate detection, particularly in heterogeneous cell populations [12, 53]. Hence, computational methods for standardizing stain coloration could be applied in future studies [49, 98]. Automated image analysis is sensitive to threshold settings and parameters used in QuPath and may misidentify cell boundaries or fail to reliably discriminate weakly positive from negative cells, especially in tissues with irregular staining or overlapping cells [97]. Biological variability, including tissue heterogeneity and processing-induced artifacts, can introduce additional variability [8, 63]. Moreover, standardizing image contrast and staining intensity between probiotic and control groups remains challenging, and operator-dependent selection of regions of interest or parameter adjustments can introduce bias [49, 94, 102]. These factors should be considered when interpreting morphometric results and comparing cellular marker expression between experimental groups.

## Concluding Remarks

In conclusion, *Saccharomyces cerevisiae* (SC) supplementation demonstrates immunomodulatory effects in broiler chickens, characterized by reduced splenic lymphoproliferative activity (decreased CD3^+^ and CD4^+^ cell density) and diminished positivity percentages for CD3^+^, CD4^+^, and CD25^+^ cell populations.

These findings indicate that SC may exert modulatory activity by decreasing antigen presentation and lymphocyte proliferation in splenic tissue, potentially promoting immune tolerance and influencing cellular redistribution. While the observed reductions in CD3⁺, CD4⁺ cell populations could be consistent with an anti-inflammatory profile, this interpretation should be made cautiously. Alternative explanations, such as immune tolerance, redistribution of immune cell subsets, or technical factors including variation in staining thresholds cannot be excluded. Further studies are required to elucidate the underlying mechanisms and confirm the biological relevance of these changes.

The implementation of digital pathology enabled precise morphometric quantification of immunohistochemical markers, providing both whole-slide digitization and automated analysis of cellular expression (positivity) and quantity (density) for each antigen-specific population under investigation, while ensuring reproducibility under identical parameter settings for the positive cell detection method of each IHC marker.

These results could support *Saccharomyces cerevisiae* supplementation as a viable antibiotic alternative in poultry production, offering immunomodulatory benefits while maintaining methodological precision in immune cell evaluation.

## Data Availability

No datasets were generated or analysed during the current study.
